# Technology-Based Early Warning Systems for Bipolar Disorder: A Conceptual Framework

**DOI:** 10.2196/mental.5798

**Published:** 2016-09-07

**Authors:** Colin Depp, John Torous, Wesley Thompson

**Affiliations:** ^1^Stein Institute for Research on AgingDepartment of PsychiatryUniversity of California, San DiegoLa Jolla, CAUnited States; ^2^VA San Diego Healthcare SystemSan Diego, CAUnited States; ^3^Beth Israel Deaconess Medical CenterDepartment of PsychiatryHarvard Medical SchoolBoston, MAUnited States; ^4^Research Institute for Biological Psychiatry, Mental Health Centre Sct. HansCopenhagenDenmark

**Keywords:** psychiatry, mHealth, prevention, technology, psychotherapy

## Abstract

Recognition and timely action around “warning signs” of illness exacerbation is central to the self-management of bipolar disorder. Due to its heterogeneity and fluctuating course, passive and active mobile technologies have been increasingly evaluated as adjunctive or standalone tools to predict and prevent risk of worsening of course in bipolar disorder. As predictive analytics approaches to big data from mobile health (mHealth) applications and ancillary sensors advance, it is likely that early warning systems will increasingly become available to patients. Such systems could reduce the amount of time spent experiencing symptoms and diminish the immense disability experienced by people with bipolar disorder. However, in addition to the challenges in validating such systems, we argue that early warning systems may not be without harms. Probabilistic warnings may be delivered to individuals who may not be able to interpret the warning, have limited information about what behaviors to change, or are unprepared to or cannot feasibly act due to time or logistic constraints. We propose five essential elements for early warning systems and provide a conceptual framework for designing, incorporating stakeholder input, and validating early warning systems for bipolar disorder with a focus on pragmatic considerations.

## Introduction

The potential for technology to facilitate “early warning systems” for bipolar disorder has been described for several decades [1-3]. A number of reports have identified the existence of near-term precursors of mood episodes [4-6]. These factors can be internal to the patient's “warning signs” (eg, disruptions in sleep/wake cycles) or external factors or “triggers” (eg, life events) that are associated with increased risk for worsening course. It is apparent that warning signs are highly varied across individuals, although some data suggests they are consistent within the same individuals over time (so called relapse signatures) [7]. Due to the importance of early warning signs, skills in monitoring and developing action plans to respond to them are a core element of many of the psychosocial interventions for bipolar disorder [8].

A promise of high frequency data collection agents, such as mobile health (mHealth) technologies, is that potential warning signs and outcomes can be prospectively and concurrently monitored. Moreover, predictions about the near future could be more accurate if based in part on accumulating knowledge about a given patient, rather than upon more static risk factors that frequently fail to predict near-term trajectories for a given individual. A variety of passive and active technologies have been piloted in bipolar disorder to this end [9-12], and while the evidence base is limited and technologies seem better at monitoring depressive than manic symptoms [12], there is some convergence of opinion that electronic self-monitoring will become increasingly used in the management of this illness.

Within a high frequency self-monitoring framework, an early warning system might gather high frequency data on both predictors (eg, sleep/wake cycle) and outcomes (eg, onset of mood episodes), identify when changes in sleep/wake cycles that previously heralded a mood episode occurrence, and subsequently deliver an alert or intervention targeted to the early warning sign in a timely fashion. Existing applications of early warning systems are already part of daily life (eg, credit card fraud monitoring). Despite the potential of early warning systems, there are a number of challenges to validation and also potential harms. Problems may arise, both if early warning systems produce incorrect predictions and lead to unnecessary distress or resource inefficiency, as well as if predictions are accurate but patients or other stakeholders are unprepared or ill-equipped to act on warnings. This paper proposes a framework for developing, validating, and implementing early warning systems with technologies that collect intensive longitudinal data in bipolar disorder.

## Proposed Architecture of Early Warning Systems

We propose a conceptual framework for design elements of an early warning system. The following are the five elements of an early warning system (Table 1): (1) a *platform* or networked collection of platforms that enables frequent data collection (eg, mobile phones, sensors), (2) *inputs* that produce intensive longitudinal data (eg, repeated self reports, collection of behavioral data such as voice, sleep, activity) and *outcomes* that could either be externally monitored events, states, or other streams of intensive longitudinal data, (3) *predictive statistical analytic* methods that link inputs with outcomes, (4) *decision rules* that determine the thresholds and actions according to the prediction based on a time horizon between the timing of the input data and the outcome that are predicted, and (5) *preventative feedback*, which may vary by content, format, delivery method, and intended audience.

**Table 1:** Proposed components of an early warning system, selected techniques, and research gaps.

Component	Selected resources	Research gaps
Platform	Mobile phone apps; text messaging; home-based telehealth; wearables	Best practices for long-term adherence and engagement; effective integration of multiple platforms; user preferences and methods for granular control of transmitted information
Inputs and outcomes	Patient reports of mood and related risk factors; passive activity/location sensing; passive metadata sensing; serial physiological sensors	Predictive validity of near future and rare events; optimal data capture frequency and duration; interpretability of passive data for warning systems
Predictive analytics	Linear and non-linear models for intensive longitudinal data; machine learning; within-sample and out of sample validation techniques	Integration of within-person and between-person data to inform predictions; integration of high dimensional variable frequency data; utility of non-linear, complex models in practicable early warning systems; validation metric criteria
Decision rules	Clinically important thresholds; empirically defined thresholds based on classification models; recursive analyses to define earliest detectable change in risk at threshold	Developing interpretable decision rules based on multiple inputs or interactions; updating decision rules based on accumulating data within patients
Feedback	Multiple communication platforms with which to alert stakeholders; elements of evidence-based behavioral change content developed for risk factor self-management in bipolar disorder	Optimization of feedback messaging to enhance self-efficacy and health protective behavior; identification of potential patient and other stakeholder’s experience of adverse impact of forewarnings; research methods for quantifying the impact of individual feedback strategies; impact of feedback messaging tailoring by mood state, patient preference, and/or severity of risk

### Platform Considerations

In considering the elements in Table 1, there is evidence to support the feasibility and acceptability of data capture platforms that use computer [13], mobile texting [14], wearable technologies [15], and mobile phone apps in monitoring mood symptoms and other inputs in bipolar disorder [12]. Platforms may increasingly combine passive sensor and device metadata (eg, duration and timing of calls) with patient reported data. Advancements in software platforms for data collection are beyond the scope of this paper, but increasingly toolkits are available to generate apps that provide the interactive data collection framework on mobile devices such as with Apple Research Kit and Android Research Stack.

For the purpose of early warning systems, key requirements for platforms would be the availability of online statistical analysis as data is accumulated, which typically and essentially if data from other individual’s are used in prediction models, would involve transmission of data from the device to a server for online analytics. Efficiency of transmission and interoperability of information sources gathered from the device or multiple devices remains a challenge that is especially pressing. Many of the electronic self-monitoring tools for bipolar disorder reviewed by Faurholt-Jepsen et al [12] were validated over periods of 3 months, which then necessitated that the predicted outcome occur during that span, resulting in a low probability of capturing multiple episode-level relapses for most patient populations. Ideally such systems were to be developed to capture more infrequent events over longer periods, and it is unclear what platform-related factors contribute to longer-term engagement (>3 months) in data collection. There is some suggestion that long-term engagement might be enhanced through interventions that promote continued use and perceived benefit, as well as the use of an individual's own device rather than study phones.

Since data is being transferred, which could include personally identifying information (eg, phone numbers, location) as well as information about symptoms and other potentially sensitive information, mHealth platforms add a risk of loss of confidentiality. A recent review found that 75% of commercially available apps purported to assist with self-managing bipolar disorder did not include a privacy policy [16]. Given that consumer buy-in for early warning systems would especially necessitate “trustworthiness” since the intent is to provide meaningful information about future personal risks, data security procedures and risk from the device and/or sever to the cloud would need to be explained to users, including the potential consequences in loss of privacy if devices are lost [17]. When asked, patients express preferences for granular control over elements of transmitted data and so apps for enabling such control, beyond just informing patients of the types of data being transmitted, may further facilitate patient engagement in early warning system design [18].

### Inputs and Outcomes

Potential inputs to early warning sign systems are far ranging and include behaviors (eg, social behaviors, substance use, changes in activity), sleep (eg, number of hours, quality), stressors, medication adherence, and affective states (eg, anxiety, irritability). These inputs measured with the device need to be validated as convergent with gold standard clinical ratings. To date, most validation studies in bipolar disorder using mobile devices have focused on the validity of mood ratings, and thus focus on the validity of measurement of the typical outcome of early warning systems rather than predictors. As reviewed recently, information technology strategies for self-monitoring indicate positive indication of the concurrent validity of aggregated momentary self-ratings of depressive mood states (although less consistently with manic symptoms) captured on mobile devices with clinician-rated data [12], with greater intra-variability than paper-and-pencil mood charts [19]. Further toward the path of an early warning system, integrated information from multiple sensors has accurately identified the presence of episodes among patients followed longitudinally [20]. It is unclear if patient reports of other inputs, such as stressors, medication adherence, or social function are associated with in-lab measures.

There are proof of concept data suggesting that passive sensor data obtained through actigraphy can individuate bipolar disorder from patients with depression and healthy controls [21]. In addition, geographic or vocal tone correlates with concurrent clinician rated mood symptoms and metadata from interactions on the device are also moderately associated with symptoms [11]. While mobile phones and their multiple embedded sensors offer new streams of data, such as call logs to understand social patterns in bipolar disorder patients, understanding the clinical translation of such data is still an area of novel research and not fully understood.

Taken together, these early stage studies indicate that mobile devices could be feasibly used to monitor at least some of the potential inputs and symptom outcomes over time. Challenges for inputs and outcomes in early warning systems are that mHealth technologies create data that is complex and unique from typical panel-type data, explained in terms of its high volume, velocity, and variety [22]. The potential to collect large amounts of data must be balanced with adherence, missingness, interoperability, and validity concerns. Studies using mobile data collection have repeatedly shown that user adherence is not perfect and participants either completely stop using these devices or provide data less frequently than planned. Indeed, missing data has been attributed to a lower than anticipated accuracy in a pilot study of the prediction of relapse in bipolar disorder using actigraphy [23]. Adherence issues may require robust statistical models for imputation [22] and may also be modeled as an additional input. Other issues with collecting these new data steams the inter-stream and temporal correlation that will require partnerships with data science experts to fully utilize and best understand the potential of real-time self report, behavioral, and physiological data [24].

Moreover, some critically important outcomes and potential new data sources would seem highly relevant to early warning systems but have received little research. In particular, suicidal ideation and risk of self-harm has been effectively assessed over time in non-bipolar samples with attention to affective states prior to thoughts [25]. It is notable that patients may actually be more open and willing to disclose suicidal thoughts to a digital device than in person to a clinician. Some people now also report suicidal thoughts and symptoms of their mental illness to online forums and their digital messages provide a new source of data on suicide risk (eg, TalkLife), and a number of studies have linked risks of suicide from texts extracted from social media and blog posts [26]. Data collection in the context of an intervention that directly heralds suicidal behavior raises the need for a robust clinical response framework [27]. As detailed elsewhere, there are a number of ethical and privacy pitfalls in incorporating social media data into to clinical decision making [28], and providing patients with granular control over the inputs to early warning systems would seem to be especially applicable to social media.

### Application of Prediction Models

Prediction models link input data and subsequent outcomes. A variety of emerging methods are used to model intensive longitudinal data and predictive analytics applied to these complex data that have varying rates and structures. Techniques such as data mining, machine learning, and probabilistic modeling have been employed to make predictions about the future. While a discussion of these individual techniques is beyond the scope of this paper (see [29]), effective early warning systems would require automating analyses and responsive updating predictions based upon incoming data.

Irrespective of the statistical technique applied, validation of an early warning sign system would center on the accuracy and preventative utility of prediction. There are several steps beyond identifying a group-level association between T_-1_ predictor and T_-0_ outcome. In terms of raising the clinical utility of predictions, the standard for gauging the usefulness of prediction would additionally include (1) how accurate the prediction is for a given individual; (2) how interpretable the predictions are in the formation of decision rules; and (3) how much lead-time is provided in which alter the course of the predictor, if alterable.

Complex time series models have been applied to understanding the dynamics of mood course in bipolar disorder, focusing on the potential for non-linear chaotic or latent approaches to modeling state shifts in the context of intra-individual noise [30-33]. Here we focus on potentially more accessible linear models. In linear models, estimates generated from training data in ordinary least squares regression frequently result in overfitting, with validation samples highly likely to perform worse than the training set. Techniques such as penalized regression can reduce the likelihood of overfitting [34]. Given that most samples are likely to be small at proof-of-concept stages, validation with independent samples is typically impossible. Within sample alternatives to validation include leave-one out validation [35].

Such models gauge the accuracy of predictions in samples with and without predicted outcomes. However, this conflates potential individual differences in the predictor-outcome relationship. In repeated measures designs, case-crossover analyses can examine the association between predictors and outcomes within patients when outcomes occurred and earlier or later times when outcomes were present [36]. This approach could help to identify the within-person sensitivity of predictors.

As an example, Thompson et al [37] employed functional data analysis to identify prospective time-lagged relationships between changes in daily-assessed negative and positive affect and observed the emergence of suicidal ideation two to three weeks later. Here, changes in affect can be seen as potential early warning signs of increases in suicidal ideation. The authors found that accuracy of the association between negative affect and suicidal ideation observed two to three weeks later was quite accurate (88% sensitivity and 95% specificity), and more accurate than predictions that relied on baseline levels of suicidal ideation. In this way, indication of a time lagged relationship between inputs and outcomes suggests a potentially causal relationship and also provides a window of time between input and outcome in which to deliver feedback regarding an impending risk of suicidal ideation and possibly attempt to alter the early warning sign.

### Creation of Decision Rules and Time Horizons

With validated data and prediction rules, a combination of clinical acumen, patient preferences, and data driven models will be necessary to create decision rules that can guide clinical interventions. While some of these decision rules may be more straightforward, such as a link between cessation of a medication and risk of switch into mania, others rules will be more complex. For example, considering insomnia as a symptom of bipolar disorder, applying the right intervention at the right time and for the right patient will likely be a personal, dynamic, and varied response with no clear-cut point or binary decision. Such decision rules can be developed empirically and a first step will require determining how sensitive and specific the input is in predicting subsequent outcomes, such as by use of a penalized regression technique. Accuracy can be judged by a metric (eg, area under the curve, AUC) generated by leave-one-out validation, and can be compared to baseline estimates of risk (eg, most recent suicidal ideation rating), and/or relative to prior windows of time that did not result in suicidal ideation, as in the case-crossover method. If sensitive and specific to the criteria listed above, sensitivity analyses to determine when prior to the outcome the earliest detectable increase in the outcome occurs (eg, examining the accuracy of prediction by censoring predictors within the span of 1-3 weeks prior to the outcome), based on an assumption is that time-varying predictors increase in accuracy the closer in time to the outcome. The identification of the time of earliest detection would be determined by comparison of accuracy when moving a stable window of information back in time to when the prediction accuracy falls below a clinical metric of accuracy (eg, AUC < 0.80) or a patient-preferred tolerance. To generate cut points, regression tree methods can then be used to identify the levels of the input that would be associated with the best fitting model (ie, with the largest AUC). These cut points can then be used to form decision rules and updated with machine learning algorithms in real-time, and made more precise to the individual weighting information from the individual, relying increasingly on individual data over that from other individuals’ data. Multiple inputs and interactions among inputs may also create even more powerful models, yet with the tradeoff of greater complexity in implementation, and more importantly, diminishing interpretability toward targeted feedback, described next.

### Feedback and Clinical Application

The empirical understanding of best practices in the feedback component of early warning systems is in its infancy. Since early warning systems will involve communication around a future probabilistic risk, there is substantial evidence to suggest that people may variably interpret or misinterpret risk communication [38]. As such, the content and form of the feedback aspect of early warning systems is critical, and one in which perspectives of providers and patient stakeholders are essential for understanding how best to communicate risk while mitigating potential adverse impacts, and maximizing tolerability, usefulness, and timeliness.

Feedback from early warning systems may be more effective if it extends beyond simply notifying patients, clinicians, and stakeholders of an impending risk. There is substantial evidence that to change behavior in response to a future risk, the arousal of fear or threat is insufficient and possibility counterproductive, particularly among people with low self-efficacy to make changes [39]. For example, an early warning system describing a risk for a near-term manic episode might arouse fear about the consequences of mania, but without specific instructions about how to avert the manic episode, respondents may discredit the message [40]. A related concern is that, if predictions derive from multiple sources of inputs including sensor data, it might be difficult to directly interpret; the drivers of risk may be somewhat of a “black box” and may not translate to a greater understanding of the illness course or actionable steps to mitigate risk. Clinicians may face the same conundrums with acting on the content of warning messages as do patients, additionally with the concern of balancing time allotted to care for patients with current *known* risks versus future *possible* risks.

There are several potential approaches to enhancing the effectiveness of warnings derived from the broader health psychology literature [41]. Messages may have greater impact if they can winnow out actionable predictors that are meaningful to the individual, emphasizing one behavior change at a time. Once the target behavior is identified, messages may be more likely to lead to changes in behavior if they (1) focus on increasing self-efficacy to act and the benefits of acting rather than evoking fear about the risk of harm [42]; (2) identify strategies that require low effort or facilitate reducing effort such as through implementation intentions [43]; and (3) provide affirmations of the individual prior to requiring the processing of threat, if delivering information about the threat is necessary [44]. It is unknown how variation in mood state may impact the receptiveness of different warnings, but it is technically feasible for messages to be tailored to the mood state of the individual to maximize persuasiveness.

Designing the feedback element of early warning systems should involve the incorporation of stakeholder perspectives, both patients and clinicians, which can occur at multiple levels. At a broader level of intervention *design*, participatory design methods have been evaluated in mental health technology-focused projects [45]. Although it is unclear if participatory approaches yield greater uptake and impact than researcher-centered programs, participatory methods typically include design cycles in which user input is sought at the outset and after multiple phases of development, from inception to deployment. For early warning systems, patient input about the interpretability and timing of warnings along with content to accompany those warnings would be essential, as is clinician input about the liabilities associated with warnings. At the narrower level of the early warning system design, there are a number of methods for incorporating patient preferences and language into feedback. For example, patients may create their own messages to accompany warnings for future display [9]. In our prior work, patients generated statements that they would like to tell themselves or adaptive behaviors they could employ in depressed or manic mood states, which were then presented to them later through the device upon reporting such a state. Many other means providing user control via mHealth are possible, such as the frequency, timing, and communication channel of feedback, as well as delineating whether and which other people are also recipients.

Within early warning systems, there will be practical challenges to understanding the impact of various feedback strategies. As with all prevention research, it is challenging to determine whether specific elements of early warnings systems have an effect at the individual level, given that it is impossible to know if future threats would have occurred without such a warning. As such, understanding the patient and message variables of effective risk communication using prediction models may be best advanced in experimental studies with proxy measures of target behaviors rather than episodes, at least in initial development stages.

## Discussion

### Summary

Much work is required to make early warning systems accurate, useful, and safe for people with bipolar disorder and their care teams. Nonetheless, there have been dramatic advances in technology-based data capture, statistical prediction analysis, and risk communication that together form the ingredients of a variety of early warning systems for bipolar disorder. Many such systems have been proposed and are in the proof-of-concept stages of development, and soon will be available to consumers and clinicians. These systems may make it possible for patients to better understand and manage bipolar disorder, avoid or forestall illness exacerbations, and minimize disruptions in social and productive roles associated with illness exacerbations.

### Conclusion

We have described a basic framework for designing interventions alongside patients and clinicians, and validating and evaluating such systems. In particular, we caution that early warning systems must empower patients to make changes rather than to simply sound alarms. We hope that this paper stimulates future development in this exciting area.

## Abbreviations

AUC: area under the curve
mHealth: mobile health
